# Supplementary figures and images for: Prediction of PIK3CA mutations from cancer gene expression data
Source: PLoS One. 2020 Nov 9;15(11):e0241514. doi: 10.1371/journal.pone.0241514 (PMC7652327; doi:10.1371/journal.pone.0241514)

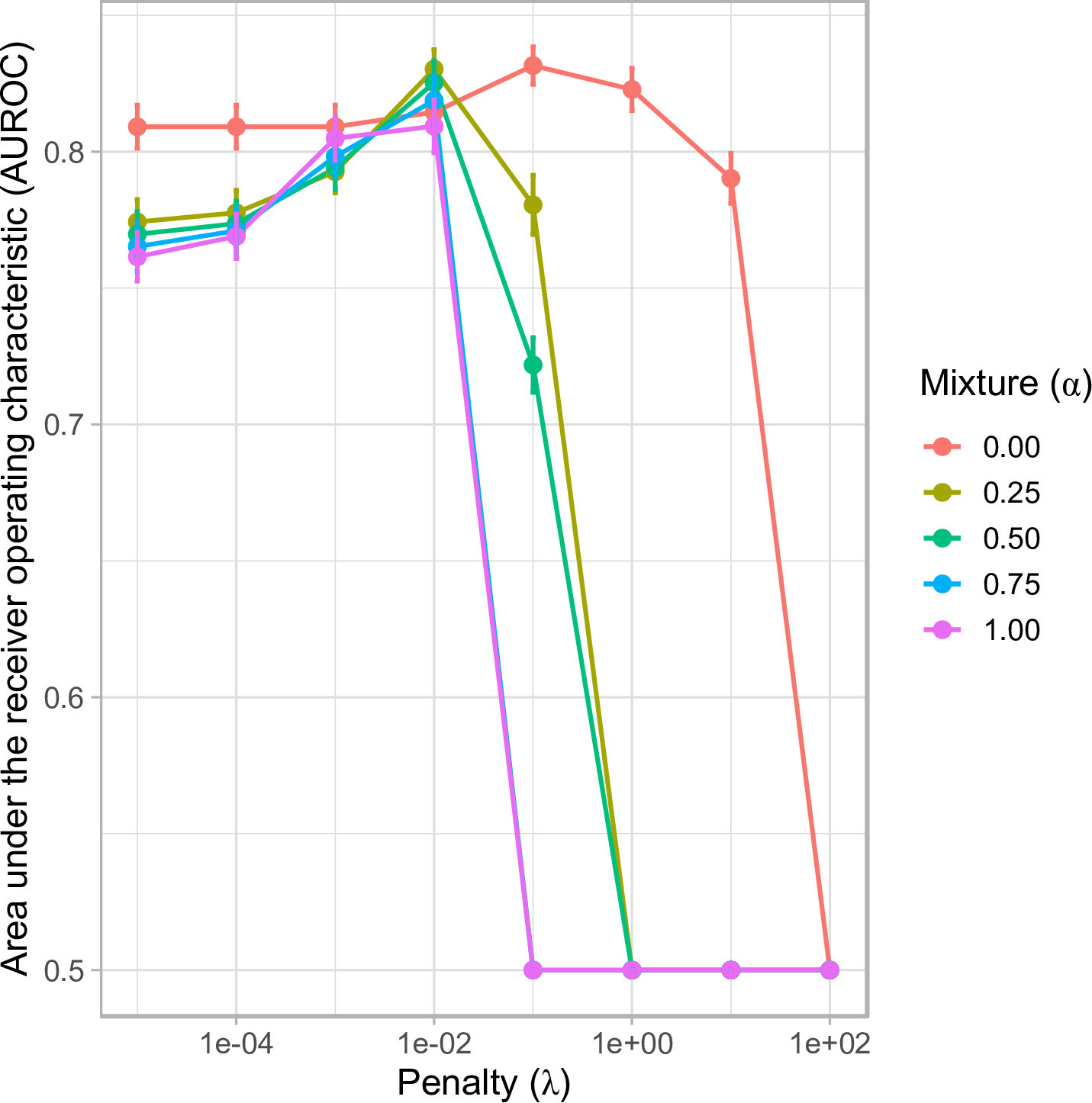

Supplement: S1 Fig — The x-axis is a penalty scaling parameter: λ {10−5, 10−4,10−3,10−2,10−1, 100}, color is mixture hyperparameter of penalty function: α {0.0, 0.25, 0.5, 0.75, 1.0}. y-axis is estimates of area under the receiver operating characteristic (AUROC) using 10-fold cross-validation resampling. (TIF) [file pone.0241514.s002.tif]
