# Supplementary material for: Prediction of PIK3CA mutations from cancer gene expression data
Source: PLoS One. 2020 Nov 9;15(11):e0241514. doi: 10.1371/journal.pone.0241514 (PMC7652327; doi:10.1371/journal.pone.0241514)
Supplement: Attachment — Submitted filename: Response-to-Reviewers.pdf [file pone.0241514.s003.pdf]

# Response to Reviewers

Dear, Editor-in-Chief, PLOS ONE

Thank you for considering our manuscript entitled "Prediction of PIK3CA mutations from cancer gene expression data (PONE-D-20-22669)." We are really happy to hear that the manuscript is considering for publication with a revision. We considered all suggestions that were raised by the reviewers and tried to answer to all queries to the best of our capability. In our annotated version, we indicated what we responded to reviewers' suggestions by using Track Change function with marginal notes and symbols (e.g., R1-1 indicates response to comment #1 of Reviewer #1). Our specific responses are as follows:

Comments to the Author

1. Is the manuscript technically sound, and do the data support the conclusions?

The manuscript must describe a technically sound piece of scientific research with data that supports the conclusions. Experiments must have been conducted rigorously, with appropriate controls, replication, and sample sizes. The conclusions must be drawn appropriately based on the data presented.

Reviewer #1: Partly

Reviewer #2: Yes

2. Has the statistical analysis been performed appropriately and rigorously?

Reviewer #1: I Don't Know

Reviewer #2: Yes

3. Have the authors made all data underlying the findings in their manuscript fully available?

The PLOS Data policy requires authors to make all data underlying the findings described in their manuscript fully available without restriction, with rare exception (please refer to the Data Availability Statement in the manuscript PDF file). The data should be provided as part of the manuscript or its supporting information, or deposited to a public repository. For example, in addition to summary statistics, the data points behind means, medians and variance measures should be available. If there are restrictions on publicly sharing data—e.g. participant privacy or use of data from a third party—those must be specified.

Reviewer #1: Yes

Reviewer #2: Yes

4. Is the manuscript presented in an intelligible fashion and written in standard English?

PLOS ONE does not copyedit accepted manuscripts, so the language in submitted articles must be clear, correct, and unambiguous. Any typographical or grammatical errors should be corrected at revision, so please note any specific errors here.

Reviewer #1: Yes

Reviewer #2: Yes

5. Review Comments to the Author

Please use the space provided to explain your answers to the questions above. You may also include additional comments for the author, including concerns about dual publication, research ethics, or publication ethics. (Please upload your review as an attachment if it exceeds 20,000 characters)

Reviewer #1:

Comment 1

This is a regulated regression analysis to find out the most significant variable(s) and that is kept in the final model. This is a very concise article and lack of detail methodology.

Response

Thank you for your constructive feedback considering the lack of detailed methodology. TCGA is a widely used public data of cancer genomics. The detail of the TCGA pan-cancer data is described in the reference. For the method of prediction modeling, we tried to follow guidelines for developing and reporting machine learning predictive models in biomedical research. We add a supplementary figure to help understand hyperparameter tuning.

Luo, Wei, Dinh Phung, Truyen Tran, Sunil Gupta, Santu Rana, Chandan Karmakar, Alistair Shilton, et al. "Guidelines for Developing and Reporting Machine Learning Predictive Models in Biomedical Research: A Multidisciplinary View." Journal of Medical Internet Research 18, no. 12 (2016): e323. https://doi.org/10.2196/jmir.5870.

Comment 2

It has been known for quite some times that PIK3CA GOF mutation is very common (in fact next to TP53) in solid tumors. It is also known that PIK3CA is very much related to PTEN and IGF1R signaling. The finding is not new and the rationale for this article is not very clear.

Response

Thank you for your opinion. Our study aims to build the PIK3CA mutation prediction model not to search for important variables. Because the penalized logistic regression model is highly interpretable, we were able to find significant variables like IGF1R and PTEN. But this findings of significant variables is not the primary purpose of this study. The purpose of this study is to investigate the *PIK3CA* mutation prediction performance of machine learning models. The purpose of the study is further described in the manuscript.

Comment 3

More importantly, this type of article is not suitable for PLOS ONE audience. Authors may consider to some bio-informatics or bio-statistics journal.

Response

Thank you for your suggestion. Since machine learning modeling is complex and has begun to be widely used relatively recently, the audience may lack an understanding of detailed methods. However, our study used a widely used data set (TCGA) and modeling framework (R tidymodels package). We believe our research will benefit audiences interested in applying machine learning to patient care. We also believe that publishers targeting a broad audience are publishing predictive model studies using machine learning.

Reviewer #2:

Comment 1

The authors present a succinct study on the prediction of PIK3CA mutations from gene expression data. This study applies an elastic net penalized logistic regression classifier to the cancer genome atlas (TCGA) pan-cancer gene expression dataset, a method that was previously established for detecting RAS pathway activation. The methods used and the results presented in the figures appear to be appropriate for the work performed. Both the AUROC and AUPRC demonstrate predictive performance well above baseline. Limitations of the approach used were also appropriately discussed.

Response

Thank you for your opinion.

Comment 2

It may be questionable why PIK3CA mutation prediction from mRNA expression is useful when targeted sequencing panels can assay these mutations directly, but it has been proposed elsewhere that clinical transcriptomics may add important functional or phenotypic information.

Response

As you pointed out, the clinical utility of PIK3CA mutation prediction from mRNA expression is unclear because most direct genomic tests are more specific and sensitive than predictive models. Our prediction model is not an application that is immediately applicable to a cancer patient for detection of PIK3CA mutation. It is not known how it will be used, but finding out the mutation prediction performance using gene expression data could play a role in advancing machine learning to be helpful in patient treatment. We discussed further the limitations of this study in the manuscript.

Comment 3

Overall this work demonstrates that machine learning approaches can predict PIK3CA mutation status from gene expression data with a reasonably good level of performance.

Response

Thank you for your opinion.

Thank you again for reconsidering our manuscript. We think we did our best to revise faithfully our previous manuscript in line with the indications that were raised by the reviewer. We hope the revised version would meet with your, and the reviewer's approval and be finally accepted by the PLOS ONE.

With best wishes and respectfulness, Youn Soo Lee, MD
